# Advances in assessing the volume of odontogenic cysts and tumors in the mandible: a retrospective clinical trial

**DOI:** 10.1186/1746-160X-9-14

**Published:** 2013-04-20

**Authors:** Marcus Stoetzer, Franziska Nickel, Majeed Rana, Juliana Lemound, Daniela Wenzel, Constantin von See, Nils-Claudius Gellrich

**Affiliations:** 1 Department of Oral and Maxillofacial Surgery, Hanover Medical School, Carl-Neuberg-Strasse 1, Hanover D-30625, Germany; 2 Department of Biometry, Hanover Medical School, Hanover, Germany

**Keywords:** Odontogenic lesion, Segmentation, Cyst volume, iPlan

## Abstract

**Purpose:**

To compare two methods of creating three-dimensional representations of mandibular cysts and tumors on the basis of computed tomography (CT) and cone beam computed tomography (CBCT) data.

**Methods:**

A total of 71 patients with acquired jaw cysts took part in this retrospective clinical study. CT and CBCT scans were obtained from all patients and saved in the Digital Imaging and Communications in Medicine (DICOM) format. Data were analyzed twice with iPlan software. Analysis was performed manually and using an interpolarization algorithm. The accuracy of the two methods in assessing cyst volume was compared.

**Results:**

Manual delineation did not provide more accurate results than the interpolarization algorithm.

**Conclusion:**

There are no major differences between manual analysis and analysis using the interpolarization algorithm. The use of the algorithm, however, has the advantage of rapidity.

## Introduction

Cystic and cyst-like lesions of the mandible are primarily ellipsoid, radiolucent, and clearly demarcated and may be odontogenic or non-odontogenic. Odontogenic cysts and tumors develop during or after the formation of teeth [1].

The distribution of jaw cysts according to diagnosis in a general population is: radicular cysts (RCs) 56%, dentigerous cysts (DCs) 17%, nasopalatine duct cysts (NPDCs) 13%, odontogenic keratocysts (OKCs) 11%, globulomaxillary cysts 2.3%, traumatic bone cysts (TBC) 1.0%, and eruption cysts (EC) 0.7% [2]. According to the 2005 World Health Organization (WHO) Classification of Tumors [3-5], OKCs, which were renamed as keratocystic odontogenic tumors (KCOTs), are benign uni- or multi-cystic [6], intraosseous tumors of odontogenic origin, with a characteristic lining of parakeratinized stratified squamous epithelium and potential aggressive, infiltrative behavior [7]. Although KCOTs are benign, they can be locally aggressive and tend to recur after treatment. Reported recurrence rates range from 3% to 60% [8,9]. Several long-term developmental processes take place in the maxillofacial area during the pediatric age period. These include the three-dimensional growth of the maxillofacial skeleton as well as odontogenesis of the deciduous and permanent dentition, all of which may be associated with cyst formation. During the adult age period, the permanent dentition sustains damage originating from caries and/or trauma, both of which may be associated with cyst formation.

Most cysts of the jaws are discovered incidentally on panoramic radiographs or they destroy surrounding structures and cause problems such as loosening of teeth or facial deformity. Computed tomography (CT) and cone beam computed tomography (CBCT) are useful tools for diagnosing and assessing cysts [10]. CT and CBCT, however, allow cysts to be evaluated only on two-dimensional cross-sectional images [11]. These images are then used for planning the surgical procedure [12]. Three-dimensional images provide a detailed representation of cysts in bone tissue and the involvement of surrounding structures such as tooth roots and nerves. For this reason, they allow surgeons to accurately plan surgical management. In this study, we compared two methods of creating three-dimensional representations of cysts on the basis of CT and CBCT data using iPlan software [13].

## Material and methods

A total of 71 patients took part in this study, which was approved by the local ethics committee. Of these, 17 patients had two cysts. All cysts were measured using the aforementioned techniques. Measurements were performed three times in order to minimize the risk of errors. The first method involved measuring the cysts three times in the axial plane using the interpolarization algorithm. The second method involved outlining the cysts in all three planes. All 71 patients were included in the statistical analysis. In patients with two cysts, the smaller cysts were included. The local ethics committee has been informed and had no concerns for the planned investigation.

### Measurement protocol and workflow

CT and CBCT images were obtained from all patients in order to visualize the anatomy of bony structures and the morphology of the cyst. These images served as a basis for assessing mandibular cyst volume.

The resulting DICOM data were transferred to iPlan planning software (iPlan 3.0, Brainlab®, Feldkirchen, Germany). Since further analysis and a better understanding of the clinical condition required a symmetric view of the data and the three-dimensional reconstruction images, CT and CBCT slices were oriented in the Frankfort horizontal and mid-sagittal (FHMS) planes in the axial, coronal and sagittal dimensions.

Once the data transfer was completed, anatomical structures and target cysts were segmented from the images. An automatic atlas-based algorithm was used for the segmentation of anatomical structures. The main purpose of this algorithm was to detect correspondences between atlas or template images and patient images. The structures are segmented automatically by elastic deformation of the templates to match the atlas images to a patient's images [14]. We thus obtained three-dimensional segmented images of the anatomical structures in the regions of interest and then used two different methods for calculating cyst volumes.

The first method involved manually outlining the cysts on every fourth axial slice. An interpolarization algorithm was then used to determine cyst volumes. The second method involved outlining the cysts in the axial, coronal and sagittal orientations. Since a cyst is not a normal anatomical structure and therefore cannot be automatically segmented using an atlas, the cysts were sliced manually with different brushing tools.

### Statistical analysis

The results for 71 patients (one cyst each) were analyzed. The Bland-Altman plot, which is a method of data plotting used in analyzing the agreement between two different assays, was used for comparing the methods [13]. Agreement can also be assessed on the basis of repeated measurements. A Bland-Altman plot was created using three different colors, which allow the different measurements for every patient to be distinguished. During a Bland-Altman analysis, it is common to compute the limits of agreement, which are specified as bias ± 1.96 STD (least squares mean ± 1.96 times the standard deviation of the differences). For a calculation of the limits of agreement, the variances and interaction of the methods must be determined.

A difference estimator is needed for a comparison of the methods. The null hypothesis is that there are no differences between the two methods. As recommended by Bland and Altman [13], a mixed model can be used which is based on repeated measurements. The difference estimator, the standard error, the 95% confidence interval, and the p-value are given in Table 1. The calculated least squares mean was added as a horizontal line in the Bland-Altman plot.

**Table 1:** Comparison of methods

**Comparison**	**Difference estimator**	**Standard error**	**t-value**	**p-value**	**95% Wald confidence interval for difference estimator**	
Method 1-Method 2	0.01579	0.01119	1.41	0.1627	-0.00653	0.03811

## Results

The Bland-Altman plot is shown in Table 1. The difference estimator is 0.01579 and the limits of agreement are -0.304278 and 0.335858. The variance of the first method is 0.009891. The variance of the second method is 0.01678. The covariance between the two methods is 0. This is not surprising since almost identical volume values were obtained. The overall variance of the difference between the two methods is 0.1633.

The least squares mean (0.01579) and the associated 95% Wald confidence interval are evidence that the two methods provide almost identical results (Table 1). A p-value of 0.1627 suggests that the null hypothesis is true and that the two methods produce the same results.

Our results show that accurate representations of cyst volumes can be obtained (Table 1) and that it is no longer necessary to draw a cyst in all planes.

The rapidity with which useful data are obtained makes this software a valuable tool in daily practice.

**Figure 1:**
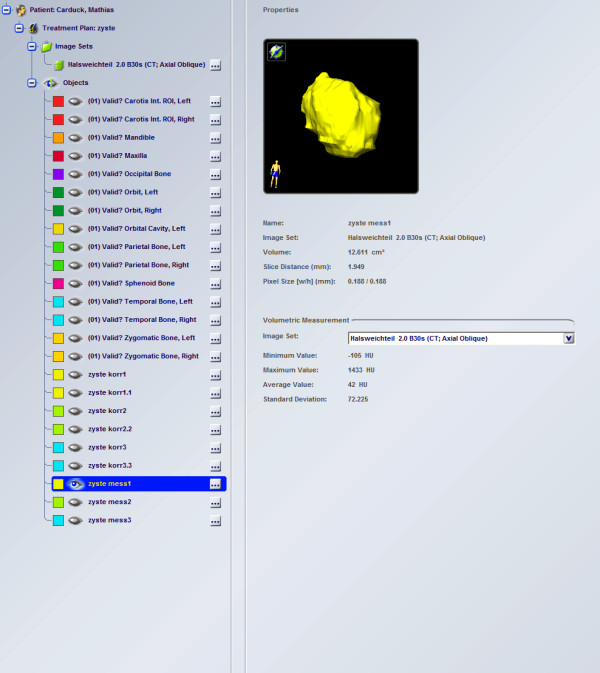
Auto-segmentation of a cyst in the mandible.

**Figure 2:**
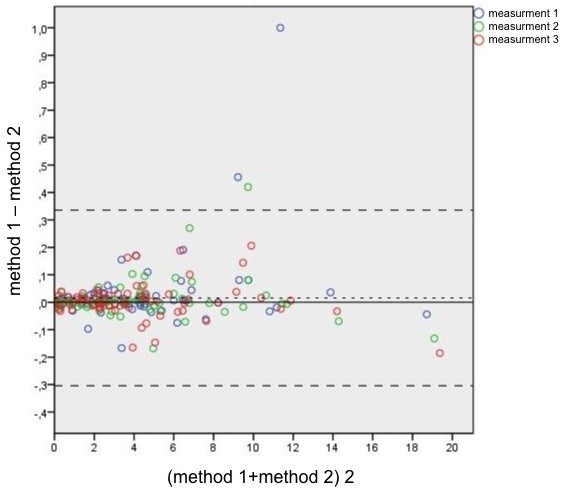
Bland-Altman plot.

The calculation of cyst volumes and the representations of cysts provide surgeons with important information about the location and orientation of cysts in bone tissue (Figures 1 and 2).

## Discussion

Zaho et al. too assessed cyst volumes. They injected saline solution into the cyst cavity and measured the volume injected. This method of measurement has the disadvantages that the solution must be injected at constant pressure and that it is unclear how much solution penetrates the cyst membrane. A further disadvantage is that the method is invasive. Computer-aided measurements of cyst volumes are non-invasive and can be performed whenever needed.

Our results show that the computer-aided assessment of cyst volumes provides information that is accurate enough to be used for preoperative planning.

The Bland-Altman plot demonstrates a cone-shaped distribution of the data and suggests that the higher the values, the larger the difference between the two methods.

**Figure 3:**
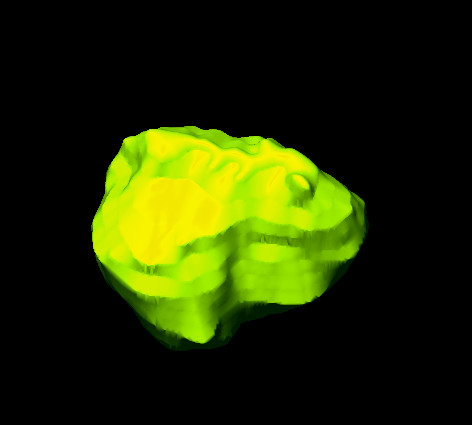
Three-dimensional image of the cyst.

**Figure 4:**
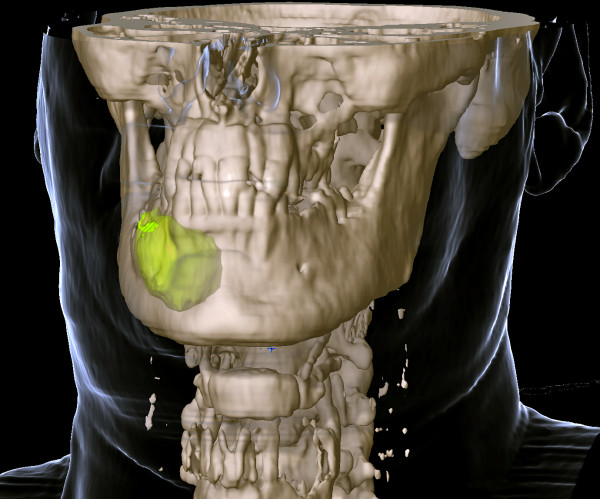
Evaluation of the cyst.

We also investigated the use of a threshold-based algorithm for assessing cyst volume. This method, however, did not provide reproducible results. Our approach of assessing cysts in three dimensions is useful for surgical planning. Adjacent structures such as teeth, the maxillary sinus and the inferior alveolar nerve can thus remain intact. The procedure is highly successful in helping decrease cyst size before enucleation and preventing extensive surgery. It is considered to be the first step in planning the surgical management of large odontogenic tumors [15]. Owing to its buccolingual position, the mandibular canal can be visualized only on axial or coronal CT scans. CBCT was found to be superior to conventional CT in detecting cortical bone involvement and delineating the mandibular canal [16]. When there is resorption of the cortex of the mandibular canal and the cyst lining is in close proximity to the inferior alveolar nerve, enucleation can damage the nerve and lead to postoperative paresthesia. A preoperative CT scan was reported to be a valuable tool for assessing this risk [17]. This is a definite indication for a three-dimensional assessment of a cyst and the segmentation of a cyst in bone tissue (Figure 1). The procedure provides surgeons with information about the total volume of a cyst and the spatial relationship between the cyst and high-risk structures in its vicinity. In addition, surgeons can evaluate the real dimensions of the cyst when removing a cyst from the bone (Figures 3 and 4).

In summary it can be said that the non-invasive determination of the volume of the lower jaw cysts is a helpful additional process in the preoperative diagnosis. The availability of software iPlan, and a good quality CT's / CBCT is a prerequisite for the implementation of the cyst segmentation, which can be recommended by the timesaving first method. Even if the calculated volume as a rational number can not replace the experience of the surgeon and the surgical field, the user is only confronted with the process of segmentation of the jaw cyst. He is inevitably confronted with the exact morphology, so that potential problems can be detected before surgery.

## Consent statement

Written informed consent was obtained from all patients. Copies of the written consents are available for review by the Editor-in-Chief of this journal.

## Competing interest

The authors declare that they have no competing interests.

## Authors’ contributions

MS, FN, MR, JL, DW, CVS and NCG contributed to the conception, design and coordination of the study. MS made substantial contributions to the acquisition of data and the preparation of the manuscript. MS drafted and wrote the manuscript. NCG and CVS revised the manuscript. All authors read and approved the final manuscript.
